# The sexuality and sexual experiences of forensic mental health patients: An integrative review of the literature

**DOI:** 10.3389/fpsyt.2022.975577

**Published:** 2022-09-26

**Authors:** Elnike Brand, Angela Ratsch, Dinesh Nagaraj, Edward Heffernan

**Affiliations:** ^1^Faculty of Medicine, The University of Queensland, Brisbane, QLD, Australia; ^2^Wide Bay Hospital and Health Service, Research Services, Hervey Bay Hospital, Hervey Bay, QLD, Australia; ^3^Rural Clinical School, The University of Queensland, Brisbane, QLD, Australia; ^4^Community Mental Health and Addiction Services, Waikato District Health Board, Hamilton, New Zealand

**Keywords:** integrative review, sexuality, sexual health, forensic, severe mental illness

## Abstract

**Introduction:**

Sexuality is an integral aspect of the human experience that defines an individual. Robust research, substantiated by the World Health Organization, demonstrates that healthy sexuality improves mental health and quality of life. Despite this level of global advocacy and clinical evidence, sexuality and sexual health as determinants of health have been largely overlooked in the mental healthcare of patients being treated under the requirements of a forensic order (forensic patients). In this review, the authors have evaluated the literature related to the sexual development, sexual health, sexual knowledge and risks, sexual experiences, sexual behavior and sexual desires of forensic patients to inform policy and clinical practice. Furthermore, the review explored how forensic patients' sexual healthcare needs are managed within a forensic mental healthcare framework. The paper concludes with recommendations for service providers to ensure that sexual health and sexuality are components of mental health policy frameworks and clinical care.

**Methods:**

An integrative review was utilized to summarize empirical and theoretical literature to provide a greater comprehensive understanding of the sexuality and sexual experiences of forensic patients. This included identifying original qualitative, quantitative, or mixed-method research, case reports, case series and published doctoral thesis pertaining to the research topic.

**Results:**

Twenty-one articles were selected for review. We grouped the review findings into three main themes: 1) Forensic patient themes, 2) Forensic mental health staff themes and 3) Forensic mental health organization themes. The review demonstrated scant information on the sexual healthcare needs of forensic patients or how health services manage these needs while the patient is in a hospital or reintegrating into the community.

**Conclusion:**

There is a dearth of evidence-based, individualized or group approaches which clinicians can utilize to assist forensic patients to achieve a healthy sexual life and it is recommended that such services be developed. Before that however, it is essential to have a clear understanding of the sexual healthcare needs of forensic patients to identify areas where this vulnerable population can be supported in achieving optimal sexual health. Urgent changes to clinical assessment are required to incorporate sexual healthcare as a component of routine mental healthcare.

## Introduction

The World Health Organization (WHO) recognizes that “sexuality is a central aspect of being human and encompasses sex, gender identities and roles, sexual orientation, eroticism, pleasure, intimacy and reproduction” (1). The WHO goes further to define sexual health as “a state of physical, emotional, mental and social well-being in relation to sexuality; it is not merely the absence of disease, dysfunction or infirmity” (1). The position of the WHO in relation to sexual health and mental health is substantiated by strong research evidence showing that positive sexual identity, positive sexual experiences, and intimate relationships improve mental health and quality of life (2, 3).

Nevertheless, despite this level of global advocacy and clinical evidence, the constructs of sexuality and sexual health as determinants of health (4) have been largely overlooked in general mental health service delivery, mental health organization design, mental health policy construction, and mental health academic research (5). Moreover, in the specific field of forensic mental health care, there is a paucity of attention.

Forensic mental health care (6) is a term that describes the provision of clinical care under the requirements of a *forensic order*, that being the outcome of a legal process in which a person has been deemed “not criminally responsible for offending behavior” due to severe mental illness (7, 67). Carroll et al. (7) report that forensic mental health patients usually require prolonged secure hospitalization where the length of stay far exceeds the requirements to manage the symptoms of SMI in this population. Accordingly, one of the goals of forensic mental health services is to enhance the capacity of patients to live successfully within the community once released from inpatient care (8–10). Ong et al. (11) indicate that most forensic patients will eventually be transferred to the community and of those, 73% will require ongoing support to enable sustained community living (12).

In meeting the goal of successfully integrating forensic patients into the community, forensic mental health services are challenged with the conflicting tasks of public protection from recidivism and ethical patient care focused upon mental health recovery principles (13). These divergent principles require a distinctively different clinical and care framework for forensic patients to that of mainstream mental health service patients (7, 14). Nevertheless, there is no conclusive evidence on how forensic patients manage their sexuality, while in a hospital or upon reintegration into the community (2, 10). Compounding this gap, is a deficit in the academic literature regarding forensic patients' sexual development, experiences, health and knowledge (15). Given that sexual health is a determinant of health generally, and the significant impact that sexuality and sexual health can have on mental health, that framework should include support for healthy sexuality and sexual health (15).

The purpose of this review is to evaluate the literature about the sexual development, sexual health, sexual knowledge and risks, sexual experiences, sexual behavior and sexual desires of forensic mental health patients to inform in-hospital and community clinical care policy frameworks and clinical practice. Furthermore, the review will explore how forensic patients' sexual healthcare needs are managed within a forensic mental healthcare framework.

## Method

### Introduction to method and methodological quality assessment

Over the past 30 years, there has been a substantial increase in the approach, volume, type, direction and complexity of mental health research. Nevertheless, preliminary literature scoping for this review indicated that both meta-analysis and systemic review were not feasible due to insufficient reports being available that would meet the rigorous inclusion criteria required for that level of analysis.

To capture the diversity and breadth of literature that has been produced, this review will utilize an integrative review methodology. An integrative review offers a comprehensive and inclusive methodology which captures theoretical literature and enables the inclusion of retrospective and prospective studies that report quantitative and qualitative research (case reports, case series), experimental (quasi and true experimental) and non-experimental (observational, descriptive, exploratory and survey) studies (16, 17). In similarity to meta-analysis and systematic review, the purpose is to appraise the quality of literature, map out central themes and related concepts and identify areas for future research (18, 19). In addition, Sparbel and Anderson (20) point out that this approach provides the opportunity to consider differing conceptual frameworks, data collection tools, data analysis approaches and synthesis while evaluating the strength of scientific evidence and assigning a weighted assessment against specific data elements. The synthesized output from this broad approach is better able to inform evidence-based practice in fields such as mental healthcare, where “health” and “care” treatment decisions can be somewhat difficult to evaluate against an “effectiveness” measure (21) obtained under meta-analysis or systematic review.

### Eligibility criteria

Articles were included if they were in peer-reviewed English language journals reporting any form of original qualitative, quantitative, or mixed-method research. We also included case reports, case series and published doctoral thesis as well as reports from the European Committee for the Prevention of Torture and Inhuman or Degrading Treatment or Punishment (CPT) (22).

Publications (including conference abstracts) that focused on (a) children under 18 years of age; b) intellectually impaired people; c) prisoners; d) non-SMI e.g., severe personality disorder as the main pathology; (e) genetics research; and (f) animal research were excluded.

### Information sources and search strategy

The electronic databases PubMed, MEDLINE, CINAHL and PsycINFO (PsycNET) were searched from 01/01/1990 until 20/04/2022. The search strategy was conducted using Medical Subject Headings (MeSH) and Descriptor of Health Science (DeCS) terms. Table 1 lists the key terms and words: “mental health disorder,” “forensic mental health,” “sexual development,” “sexual behaviour,” “sexual health” and “sexual knowledge and sexual needs.” We also included studies where participants were professional forensic mental health staff or mental healthcare services. We combined these using the Boolean AND operator adapted for each of the databases and removed duplicate publications.

**Table 1:** Search strategy.

**Concept**	**Theme**	**Terms**
**Search terms**
Concept 1	Mental health disorder	Serious mental illness; severe mental illness; major mental illness; schizophrenia; schizoaffective; psychosis; psychoses; psychotic; bi-polar; bipolar.
Concept 2	Forensic mental health	Forensic; involuntary; court-ordered; committed; mentally disordered offender; forensic mental health staff; forensic mental health services.
Concept 3	Sexual development	Sexual development; puberty; adolescence; gender identity; sexual identity.
Concept 4	Sexual experiences, behavior and desire	Sex; sexual; sexuality; sexual behavior; sexual behavior; sexual intercourse; sexual experiences, coitus; intimacy; intimate sexual needs; sexual desire; sexual attitude.

### Publication identification and quality assessment

EB, AR and DN independently screened all articles, first by title, then by abstract for eligibility and then met to reach a consensus for inclusion. Disagreements were resolved through discussion. A final selection of full-text articles was completed after further screening and consultation with EB, AR, DN and EH.

EB, AR and DN examined and assessed all retrieved publications for study quality using the Agency for Healthcare Research and Quality (AHRQ) criteria for observational studies with a consensus-based weighting score (23). Quality indicators were defined according to integrative review methods and criteria (17, 18, 21) and included (a) sample size; (b) study design; (c) attempts to control for the risk of bias; (d) use of appropriate and standardized measures; (e) use of appropriate statistics; (f) quality of the presentation of the results; and (g) generalisability. The studies were rated on a scale of 1 to 5 based on the report's quality assessment. An overall rating of good, fair, or poor was allocated to each study based on the relevance to our research question.

Data collection and data items were extracted by reviewers using an electronic (Microsoft Excel 2010) pro forma specifying the data items. The following data were extracted from each study: year published, country, participants, methods, setting, study type, aims, problem identification, method of data collection and most important findings.

## Results

### Study selection

**Figure 1:**
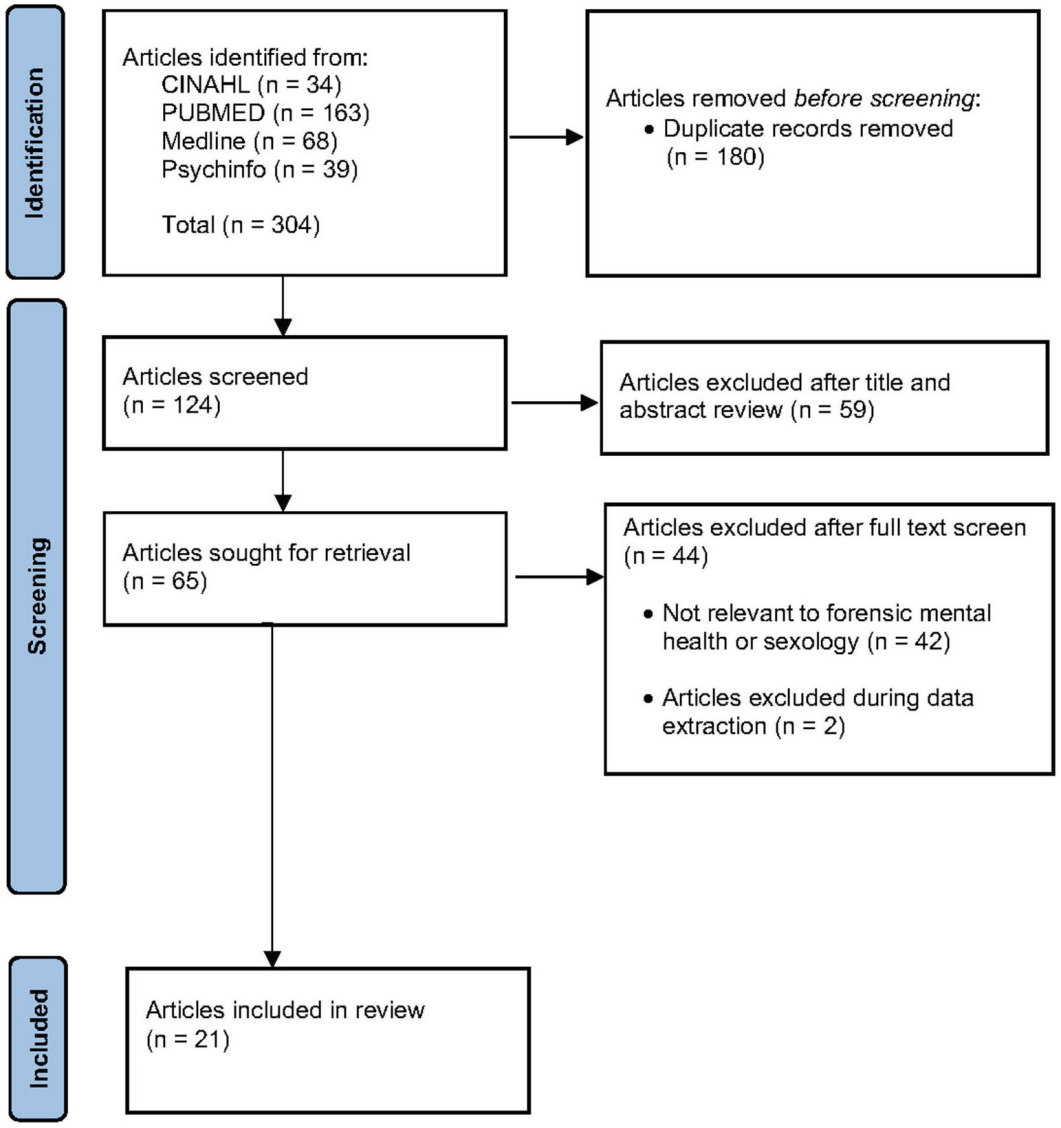
Study selection flow chart.

Figure 1 shows that the search returned 304 results, with 124 studies remaining after duplicate studies were deleted. Of these, 59 studies were excluded after the title and abstract review, and 65 were fully reviewed. From these, 21 articles were selected for assessment (Supplementary Table 1).

### Study characteristics

All 21 papers related to forensic mental health patients and comprised patients in inpatient settings and residing in community. Table 2 shows that of the 21 studies, *n* = 14 (67%) reported on data in a European setting, with just under half (*n* = 10, 48%) from the United Kingdom. Only *n* = 6 (29%) Australian specific studies were identified. While studies dated back to 2000, *n* = 12 (57%) were published in the last 10 years and *n* = 9 (44%) in the last 5 years.

**Table 2:** Summary of report characteristics.

**Data element**	**Item**	**Number of papers (*N* = )**	**Rounded (%)**
Country	Australia	6	29
	United Kingdom	10	48
	Asia	1	5
	Other European countries	4	19
Year published	2000–2005	2	10
	2005–2010	3	14
	2011–2015	7	33
	2016–2022	9	43
Type of report	Quantitative	2	10
	Qualitative	16	76
	Mixed method	1	5
	Intervention	0	0
	Systematic literature review	0	0
	Discussion paper or policy	2	10
Participants	Forensic patients = male	14	67
	Forensic patients = female	7	33
	Forensic mental health staff	8	38
Other	Protocol and documents	2	10
Setting	Forensic inpatient unit	18	86
	Forensic community	3	14

The majority of studies were qualitative (*n* = 16), with *n* = 2 quantitative studies, *n* = 1 mixed method study, and *n* = 2 discussion papers. The overwhelming setting for the studies was the forensic inpatient unit (*n* = 18) with only *n* = 3 in a community environment. Fourteen studies reported on male populations and seven studies reported on female forensic patients, while eight studies focused on forensic mental health staff. Two studies included protocols and documents only. Of the 21 reviewed articles, the quality rating against the research question was good for *n* = 11, fair for *n* = 5 and poor for *N* = 5.

#### Forensic patient characteristics

Fifteen articles included and described characteristics of forensic patients. These papers were quality rated as *n* = 4 poor, *n* = 2 fair and *n* = 9 good. Four of the papers by (24–27) were based on the same population. Accordingly, there are 12 forensic patient groups described in this literature review (Table 3).

**Table 3:** Forensic mental health patient population papers.

**Paper number**	**Author, year**	**Quality rating**	**Forensic setting**	**Total**	**Sex**	**Age**	**Diagnosis**	**Index offense**
1	(28)	Fair	Inpatient	*N =* 4	4 male	Not reported	*N =* 4 (100%) Schizophrenia	Serious criminal offending
2	(12)	Poor	Inpatient	*N =* 74	74 male	Mean 34.2 years Range 18–75 years	*n =* 44 (60%) Psychotic disorder *n =* 13 (18%) Mood disorder *n =* 6 (8%) Other mental disorders *n =* 27 (36 %) Personality disorders *n =* 9 (12.1%) Intellectual disability	*n =* 26 (35%) Aggravated assault *n =* 14 (19%) Sexual offending *n =* 11 (15%) Homicide, murder, Manslaughter
3	(29)	Good	Inpatient	*N =* 25	18 male 7 female	Male Median 27 years Range 20–33 years Female Median 39 years Range 28–42 years	*n =* 7 (28%) Psychotic disorder *n =* 14 (56%) Personality disorder *n =* 3 (12%) Combined *n =* 1 (4%) Other	*n =* 7 (28%) Homicide *n =* 7(28%) Non fatal violence *n =* 3 (12%) Sexual offending *n =* 4 (16%) Arson *n =* 4 (16%) Violent but no criminal charges and other charges
4	Mercer, (30)	Fair	Inpatient	*N =* 9	9 male	Not reported	Personality disorder	Sexual offending
5	(31)	Poor	Inpatient	*N =* 255	255 female	Mean 31.2years (SD = 10.)	*n =* 172 (67%) Mood disorder *n =* 27 (11%) Psychotic disorder *n =* 56 (22%) Personality disorder	Criminal offending
6	(2)	Good	Inpatient	*N =* 19	14 male 5 female	Range 20-55 years	A psychotic disorder or severe mood disorder	Index offense of sexual in nature were excluded
7	(24–27)	Good	Inpatient	*N =* 10	6 male 4 female	Male Mean 35.8 years Range 25-48 years Female Mean−33.8 years, Range 26 – 47 years	Not Reported	Criminal offending
8	(32)	Poor	Inpatients and community patients	*N =* 119	101 male 18 female	Mean 36 years (SD = 9.8).	*n =* 3 (3%) Psychotic disorder *n =* 35 (30%) Mood disorders *n =* 33 (28%) Personality disorders *N =* 48 (39%) Other diagnosis	*n =* 114 (96%) Criminal offenses *n =* 5 (4%) non Criminal behavior
9	(33)	Poor	Inpatient	*N =* 10	10 male	Mean 38.17 years SD (10.40)	Schizophrenia/schizoaffective disorder *N =* 10 (100%)	*n =* 2 (20%) Murder / Manslaughter *n =* 5 (50%) Assault *n =* 1 (10%) Sexual offending *n =* 1 (10%) Arson *n =* 1 (10%) Other
10	(34)	Good	Inpatient	*N =* 16	10 male 6 female	Median 27 years Range 18–36 years	Not reported	Criminal offending or pose great a risk of harm to self or others
11	Brand, 2021	Good	Inpatient and community	*N =* 4	4 male	Not reported	*N =* 4 (100%) psychotic illness	Serious criminal offending
12	(35)	Good	Community	*N =* 14	11 male 3 female	Mean 45 years, Range 25–61 years	*n =* 13 (93%) Psychotic disorder *n =* 1 (7%) Mood disorder	Serious criminal offending

Nine papers focused exclusively on forensic inpatients, one on forensic outpatients (35) and two on a combination of in and outpatients (32, 36).

All articles, apart from one (31), included male forensic patients, with five articles only including men. Six articles include female forensic patients and one paper by Dolan and Whitworth (31), focused exclusively on female patients (*n* = 255 patients). Across all articles, 298 females, and 261 male patients with a total of 559 forensic patients were included ranging in age from 18–75 years old.

Ten papers refer to the diagnosis of their participant population, with many referring to multiple diagnostic categories. Nine reported on forensic patients with a psychotic disorder, including schizophrenia and schizoaffective disorder. Of that nine, three populations only consisted of patients with a primary psychotic disorder diagnosis. Out of the total of ten papers, five articles included patients with a mood disorder, including severe depression and bipolar affective disorder. Five of the ten articles have included patients with personality disorder while one author exclusively focused on forensic patients with a personality disorder (30). Only Brown et al. (2) commented on medication, indicating that all his patients were managed by depot antipsychotic medication.

All 12 articles referred to the forensic patient's index offending. Five authors report only “serious” and “criminal” offending. Four authors described the specific offending. Specific offending ranges from arson/fire setting, violent offending, including fatal violent offending (homicide, murder, manslaughter) and non-fatal violent offending (aggravated assault, grievous bodily harm, unlawful wounding) and sexual offending. One author refers exclusively to sexual offending (30), while one author excluded any patients with an index offense of sexual offending (2). Four authors also included non-criminal but high-risk patients.

### Main themes of selected studies

For this review, we grouped the study findings into three categories according to the major topics they addressed (patient themes, staff themes, and organizational themes). Table 4 shows that forensic patient-related themes were further categorized by the identified elements of the report—the categorization was not mutually exclusive, for example, sexual health and knowledge often also pertained to sexual behavior and sexual experience, and accordingly, bracket overlap between categories was unavoidable. Nevertheless, the main sub-themes identified in the reports were: sexual development *n* = 7, sexual experiences, behavior and desires *n* = 14, and sexual health, knowledge and risks *n* = 12. Forensic mental health staff themes *n* = 13 and organization themes were discussed in *n* = 12 reports.

**Table 4:** Themes identified in the data extraction.

**Article themes extracted**	**Sub-theme**	**Number of papers (*n* = )**	**Rounded (%)**
Forensic patient themes	Sexual development	7	33
	Sexual experiences behavior and desires	14	67
	Sexual health and knowledge and risks	12	57
Forensic mental health staff themes	Not applicable	13	62
Forensic mental health organization themes	Not applicable	12	57

## Synthesis of results

The synthesis focused on the sub-themes identified from the literature: sexual experiences, behavior and desires, sexual health and knowledge and risks, and as well as forensic mental health staff and organizational aspects impacting the patient's sexual health and sexuality. Risks are discussed under sexual health and knowledge given that a lack of sexual knowledge often contributes to poor sexual health and risky sexual behavior (27).

### Forensic patient themes

#### Sexual development

Limited information was identified regarding the physical and sexual development of forensic patients even though initial forensic patient hospitalization often coincided with the critical period of development of adult sexuality and personal relationships (37). Brand et al. (36) acknowledge the bi-directional impact between mental health and sexual health and the particular importance given the young ages at which mental health problems tend to emerge and develop.

Dein and Williams (38) reported that forensic patients spend a large part of their sexual and reproductive lives hospitalized, and Rutherford and Duggan (39) reported that in the United Kingdom, 47% of forensic patient inpatient admissions exceed five or more years with 95% of these patients younger than 64 years. Lindstedt et al. (12) recognized that the impact of this disruption from community life leads to stunted development and maturation, as well as cognitive and social difficulties.

Threats and violence are common among male forensic inpatients (32), and they often have past histories of violent behavior. Previous research identified that violent men strive for power and dominance over others (40, 41). On the contrary, Searle et al. (33) identified a predominantly pro-social development framework for the representation of masculinity in forensic male patients with a psychotic illness.

### Sexual experiences, behaviors and desires

Hales et al. (29) noting that the vast majority (*n* = 24, 96%) of forensic inpatients had been sexually active at some time in their past. Lindstedt et al. (12) indicated that before admission (*n* = 44, 60%) were married, had been married, or lived in marriage-like relationships while 53% lived alone. Mercer (30) described the solitary practice of pornography and masturbation in forensic male patients in inpatient settings, while Quinn and Happell (27) reported a higher incidence of same-sex experiences amongst patients that identified as heterosexual. Despite the longevity of hospitalization, Ravenhill et al. (34), reported that patients maintain their pre-admission sexual orientation. According to Brown et al. (2), and further illustrated Brand et al. (35), Brand (36) active positive and negative symptoms of SMI, and significant sexual side effects of psychotropic medication further amplify sexual difficulties and negative sexual experiences.

Brown et al. (2) and Dein and Williams (38) recognized the difficulties forensic mental health patients have in initiating or maintaining existing relationships while Cook (42) and Koller and Hantikainen (28) also acknowledged the negative impact of a forensic sentence on relationships and Hales et al. (29) reported that no pre-admission relationship survived forensic hospitalization. The authors relate this to poor interpersonal skills, social withdrawal, lack of self-confidence, low self-esteem, guilt and feelings of shame. Challenges in socializing and communication skills were reported as a significant barriers to sexual intimacy by community forensic mental health patients (35), and this theme of a deficit in sexual intimacy was identified as a strong unmet need for most forensic patients (26) with inpatients pointing to the lack of privacy, private space and private time as the most significant barriers for them to engage in positive sexual experiences (24, 25). Patients associated this loss of privacy with the losses of autonomy, individuality and control in the forensic inpatient population (35, 36, 42).

Regardless of barriers Quinn and Happell (26), reported that forensic patients continue to have sex, often in secrecy (25). Hales et al. (29) reported that 30% (*n* = 7) of forensic inpatients indicated that they have engaged in some form of sexual activity Quinn and Happell (24), reported that forensic patients discuss sex and intimate relationships and view this as part of normal human behavior. This finding was supported by both Ravenhill et al. (34) and Hales et al. (29) who point out that forensic patients experience sexual feelings, sexual desires, interests and hopes for current and future sexual experiences and relationships. Further evidence of the “normality” of sex was reported by Brand et al. (35) with 12 out of 14 community forensic mental health patients describing a desire for sex with a regular partner. Their desired ideal of sexual intimacy ranged from having sex at least twice per week to having sex daily (an average of 3.71 times per week). In this cohort, only two of fourteen community forensic patients indicated that they were in stable relationships.

According to Koller and Hantikainen (28) as well as Brand et al. (35) forensic patients associate relationships with security, trust, care, a feeling of being loved and a deeper emotional quality. Dein and Williams (38), Hales et al. (29) and Quinn and Happell (24) reported that several forms of intimacy from affection, connection, and relationships to actual sexual activity were perceived as therapeutic and supportive by forensic patients in their recovery.

### Sexual health, knowledge and risks

Quinn and Happell (27) and Hales et al. (29) identified a lack of past or ongoing sexual education for forensic patients, however, both authors reported that forensic patients had some awareness of sexual health risks, mostly of sexually transmitted diseases. In the study by Hales et al. (29), 68% (*n* = 17) of forensic inpatients could define “safe sex,” (specified as condom use) but like Quinn and Happell (27), Hales et al. (29) also concluded that few patients engaged in safe sex practices or use condoms Hales et al. (29) reported that only 40% (*n* = 10) of forensic inpatients in a high secure hospital in the United Kingdom reported ever using condoms during sexual activity, and just 20% of these (*n* = 2) claimed regular use. Interestingly, 60% (*n* = 15) of forensic mental health patients supported the free availability of condoms in the high-security hospital and 64% (*n* = 16) indicated that they would use them if available (29).

Several authors, including Quinn and Happell (24, 26), Dein and Williams (38), Brown et al. (2) identified legitimate risks to forensic inpatients engaging in sexual behaviors, including the capacity of patients to consent to sexual intercourse, predatory sexual behaviors, sexual coercion and sexual exploitation. Brown et al. (2), Dein and Williams (38) and Quinn and Happell (26) raised the consequences of relationship breakdown and the potential risk to an already vulnerable patient's mental state. Brand et al. (35) reported in a qualitative study on community forensic mental health patients, the “fear of being hurt” in intimate relationships. Risks of unexpected pregnancies in forensic inpatients (8, 24) were also identified.

Despite largely perceiving sexuality and intimacy as positive, Huband et al. (3) identified the forensic patients' need for improving social skills and gaining sexual knowledge. Quinn and Happell (26) recognized that forensic patients required assistance with decisions around intimate sexual relationships. Brand et al. (35) in their qualitative study noted participants being able to identify the areas in which they would like to receive aid, such as knowledge about what “rights” they would have in a relationship, working on their communication skills, and regular medication reviews to maximize the treatment effect of their psychotropic medications, while minimizing side effects.

### Forensic mental health staff themes

Brand et al. (36) recognized the gap in the clinical identification and assessment of the sexuality and sexual health needs of forensic patients which in part explains the subsequent lack of management and encouragement of appropriate and safe sexual experiences in a forensic mental health setting. Several authors, including Brown et al. (2), Quinn and Happell (24), and Dein et al. (8) concluded that the most significant forensic mental health staff barriers for forensic patients' positive sexual experiences were risk-related, including risk aversion, safety fears and ambiguity in regards to patients' capacity to consent to sexual activities. Family and public disapproval and negative media responses related to forensic patients' relationships were identified as potential risks by forensic mental health staff in a study by Brown et al. (2). Additionally, inadequate staffing, lack of training (38), personal beliefs, negative attitudes (26), and avoidance of discussion in regards o sexuality and sexual health were identified as hindering patients' positive sexual experiences (24, 25).

Mercer (30) reported that male staff were sympathetic to forensic patients' use of pornography for sexual satisfaction while both Quinn and Happell (26) and Dein and Williams (38) recognized that forensic mental health staff were more accepting and provided more support to the sexual needs of long-term forensic patients as opposed to patients with a shortened admission. Dein et al. (8) indicated that no concerns were raised by staff when forensic patients engage in sexual experiences on unescorted community leave.

In a qualitative study by Brand et al. (35) community forensic patients supported the notion that mental health teams could support their sexual health. Most of these patients reported that they had no issues with being asked questions regarding their sexual health and well-being during regular reviews. Quinn and Happell (25) identified the neglect of preparing patients to engage in sexually intimate relationships in a safe and dignified manner as a major limitation in recovery-orientated forensic mental health care Brand et al. (36) point out that if the concept of a person's sexual health, as part of their holistic care is diminished, their functioning as a whole sexual being is obviated.

### Forensic mental health organization themes

The literature showed that there is a lack of comprehensive policies around sexual health within mental health settings (42–45) with several policies prohibiting or actively discouraging sexual expression and relationships (10, 38) based on risk aversion (8, 24) and restricted movement within facilities (46).

Specifically, there is no consensus on what might constitute “best practice” in forensic settings (3, 24, 34) with inconsistencies across institutions and varied attitudes and management of sexual expression and an absence of patient-centered consideration for sexual healthcare needs (47). Mercer et al. (48) and Bartlett et al. (10) both recognized that this policy position contradicts forensic settings as therapeutic and not punitive and non-discriminative. Sexuality expression and sexual and gender identity constructs were often conceptualized in terms of organizational misbehavior (27, 34) and decisions in regards to patients' sexual activities were based on professionals' personal judgment (8). Interesting, while rehabilitation guidelines failed to include support for community forensic patients to have sexual or intimate relationships (49), policies have started to include the provision of education or counseling relating to sexual and emotional relationships. Tiwana et al. (47) identified nine European countries (of which the UK was not one), that have a more positive acknowledgment of patients' rights to sexuality and relationships and allow some kind of sexual expression for forensic inpatients and especially if the permissive approach aids the therapeutic process. These countries have not reported significant problems resulting from this more patient-focused approach.

## Discussion

Sexual experiences are important components of normal human behavior (25) and their positive aspects are well recognized (50). The World Health Organization has embraced positive intimate relationships and sex with respect for dignity and privacy, as a fundamental human right (28, 51). Large nationally representative surveys on sexual health and sexuality in developed countries, such as the Australian Study of Health and Relationships (ASHR) (52) and the British National Surveys of Sexual Attitudes and Lifestyle (44) guide policymakers to develop strategies for the improvement of sexual healthcare in the community. Comparable studies in disability and prison populations also demonstrate sexual healthcare deficits (53, 54).

There is an overlap between general mental health patients and forensic patients with SMI. SMI often emerge during adolescence (55–57) with the onset often disrupting crucial sexual developmental milestones, the establishment of sexual identity and gender role perception (57), the acquirement of sexual knowledge, and establishment sexual attitudes and beliefs (58). The academic literature confirms that sexuality in patients with SMI is generally poorly assessed and infrequently explored (59–61) and has been a neglected topic of diagnosis and clinical management (62, 63). The limited literature indicates that the sexual well-being of patients with SMI is associated with complex interactions among psychological, sociological, and biochemical-pharmacological factors (64). SMI patients have a higher prevalence of sexual dysfunction (65, 66) and high-risk sexual behaviors (67–70).

There is no conclusive evidence on how forensic patients manage their sexuality while in a hospital or upon reintegration into the community (2, 10). Very limited data is available in the academic literature regarding forensic patients' sexual development, experiences, health and knowledge. No quantitative data on sexual development, experiences, health, and knowledge could be elicited despite broad literature searches. Nevertheless, most forensic patients will eventually be transferred to the community (11) and of those, 73% will require ongoing support to enable sustained community living (12).

Forensic patients face the same challenges as SMI patients with an additional unique tension between risk management and therapeutic intervention (71). For forensic patients, their SMI symptoms are often largely in remission for most of their lengthy admission while they engage in recovery and rehabilitation (6, 8, 71). In Australia, despite adopting a recovery model (72) forensic patients are confronted with sexually restrictive treatment cultures and settings and prohibitive policies that prevent their sexual health needs from being recognized or met. Specifically, a need for privacy, respect and a dignified place for patient intimacy (Quinn & Happel, 2015) is absent in policies and facilities.

The restriction of sexuality and intimacy is contrary to the ethical principle of non-maleficence (8) as well as the forensic recovery model which both supports the normalcy and legitimacy of sexual expression in human experience (34). Detention in a mental health facility as a forensic patient does not automatically imply incapacity to consent to sexual acts (26).

### Impact

Poor sexual health negatively impacts relationships, emotional and physical wellbeing (73, 74) and reduces the quality of life for patients with SMI (75–77). Furthermore, legal authority for governing (restricting) sexual expression has human rights implications (10) and even restrictions on sexual media can be interpreted as a breach of human rights (30). Koller (28) recognized that loss of privacy caused by losses of control makes autonomy and individuality impossible.

Prohibitive approaches change the patient's identity as a sexual being (8) by internalizing societal disapproval of their sexuality (2, 38). Patients disconnect with their sexuality and conceptualize sexual experiences as negative, ambiguous and threatening (2, 5). According to Quinn and Happell (24) regulation of sexual behavior serves as a barrier for the development of future relationships. Consequently, hindering patients to experience the potential benefits of intimate relationships including improvement of self-esteem, sense of belonging, mutual support, enjoyment and fulfillment from intimacy and ultimately improvement of a quality-of-life (25). In addition, deprivation and isolation from sexual activity and tactile affection correlate with several negative emotions, such as loneliness, depression, stress, and alexithymia (78).

Restrictions on sexual expression for long durations can potentially destroy existing relationships, prevent the formation of new relationships, and damage the patient's identity as a sexual being (8), and their expectations of future intimate relationships (34). Nevertheless, institutional rules force intimacy and sexual relationships into secrecy (25). Perceiving sexual behavior as misbehavior, the pervasiveness of discourses of risk, vulnerability and appropriateness (34) and the ethos of prohibition impact how rehabilitation is reviewed (2). Brown et al. (2) perfectly conceptualize the “amputated sexuality” that forensic patients develop and which becomes the framework and hindrance (34) for existing and future sexual relationships and expectations that will continue upon discharge in the community (5, 25).

## Recommendations

### Forensic patient recommendations

Forensic mental health patients would benefit from support to engage in positive and safe sexual experiences and to improve their quality of life. Bartlett et al. (10) emphasize the importance of an established secure social system in the community upon release of forensic patients, and recommends a patient-centered, individualized approach to sexual and emotional relationships. This would require addressing difficult and sensitive questions in several realms: ethics, law, social policy, clinical judgment, politics, religion, and family structures (9).

Forensic mental health staff should offer support through sexual education programs, improvement of socialization and communication skills, and address issues of internalization of stigma and self-esteem, as well as regular medication reviews (35). Sexual education should be made routinely available and incorporated as part of standard rehabilitation to all forensic patients (24, 35).

Sex education will be beneficial in addressing inappropriate sexual behavior while reducing social and sexual isolation (35). This can be further extended to the provision of healthy relationship groups (8). Provisions must be made for the availability of condoms or contraception for all patients (24).

### Forensic mental health staff recommendations

There is an absence of any evidence-based, individualized or group approaches which clinicians can utilize to support forensic patients achieve a healthy sexual life (35) and it is highly recommended that such services be developed.

Both Brand et al. (36) and Dein et al. (8) recommend the incorporating of sexuality and sexual health assessments, including assessment of patient's sexual needs and their capacity to form relationships into standard clinical assessment. Incorporating sexuality and sexual health into standard clinical assessments will contribute to supporting holistic forensic mental health recovery and improve quality of life (36) and the management of sexual health issues should form part of clinical care assessment as an essential component of overall well-being (35).

Forensic staff need to recognize the potential importance of childhood sexual abuse in their models of care given the complexity of the association between childhood sexual abuse and psychosocial needs and its impact on successful rehabilitation (31). The education of forensic staff is important to changing attitudes (8) and developing confidence and strategies to respond to normal human sexual behavior adequately and safely (25, 29). Forensic staff should offer support to, as well as provide protection for patients (26).

### Forensic mental health organizational recommendations

Respect for human dignity and safeguarding privacy, are very basic human rights that should be respected (28). However safety is always paramount, closely followed by capacity for free consent (38). Recovery-orientated care should include support for patients around their sexual relationship decisions (24) and should provide for discretion in clinical decision-making (26). Quinn therefore advocates for strategies to assist in developing confidence in responding to normal human sexual behavior as a matter of priority (25).

The development of forensic patient-specific policies that are flexible, patient-centered, pro-active, recovery orientated, and support an individualized approach to sexuality and sexual relationships would provide significant improvements (24, 26, 47). Such policies should take into account relevant legislation, social policy, clinical judgment, politics, religion, and family structures risks and the safety of patients and staff (8, 38). Uniform national policy supporting patients' sexual expression would provide significant improvements (47).

### Research recommendations

It is essential to have a clear understanding of sexuality and sexual activity of forensic patients to identify areas where this vulnerable population can be supported in achieving optimal sexual health (35).

There is a need for future studies involving forensic patients that can provide both quantitative and qualitative data in terms of sexual development, experiences, health and knowledge (15). Quinn also recognized the need for further research to consider the benefits and risks of patient intimacy and sexual relationships for long-term patients in forensic mental health settings (25).

Data obtained from these studies will be valuable to inform policymaking, guide staff education and the development interventions to ensure positive and safe sexual experiences and improvement of quality of life for forensic patients.

## Limitations

The current review presents certain limitations. First, additional terms could have been added to the initial article search (e.g., marriage^*^ or wife^*^ or husband^*^ or girlfriend^*^ or boyfriend^*^) to be more inclusive. This may have led to the identification of other relevant articles and consequently, more diverse findings. Impediments in the literature review approach centered upon the heterogeneity of forensic mental health patients under the nomenclature of SMI, the dissimilarity in reported service delivery models, the limited reporting of cohort characteristics and matching of participants, and narrow population sampling, exposure measures and statistical analysis and reporting in research papers.

## Author contributions

EB, AR, and DN independently screened all articles and then met to reach a consensus for inclusion. Disagreements were resolved through discussion. A final selection of full-text articles was completed after further screening and consultation with EB, AR, DN, and EH. EB, AR, and DN wrote the initial manuscript. AR and EH edited the manuscript. All authors have approved the final manuscript.

## Conflict of interest

The authors declare that the research was conducted in the absence of any commercial or financial relationships that could be construed as a potential conflict of interest.

## Publisher's note

All claims expressed in this article are solely those of the authors and do not necessarily represent those of their affiliated organizations, or those of the publisher, the editors and the reviewers. Any product that may be evaluated in this article, or claim that may be made by its manufacturer, is not guaranteed or endorsed by the publisher.
