# Supplementary material for: The sexuality and sexual experiences of forensic mental health patients: An integrative review of the literature
Source: Front Psychiatry. 2022 Sep 26;13:975577. doi: 10.3389/fpsyt.2022.975577 (PMC9548579; doi:10.3389/fpsyt.2022.975577)
Supplement: Supplementary file 1 [file Data_Sheet_1.docx]

| Paper  Number | Author | Setting | Participants | Methods | Quality | Rating | Findings |
| --- | --- | --- | --- | --- | --- | --- | --- |
| 1 | Koller, 2002, | Forensic inpatients, Switzerland | Forensic patients  N = 4 men | Qualitative | *** | Fair | Meaningful relationships were important, representing security and trust.  Loss of privacy and control and guilt feelings hindered relationships. |
| 2 | Lindstedt, 2004, | Forensic inpatients, Sweden | Forensic patients  N = 74 men | Multi methods | ** | Poor | Identified patients’ social disability and limited awareness and high support needs to participate in community life. |
| 3 | Hales, 2006 | Forensic inpatients, UK | Forensic patients  N = 25  n = 18 men  n = 7 women | Multi methods | **** | Good | High prevalence of previous sexual activity, new relationships and past child sexual trauma.  Reports of sexual activity on the unit, but low frequency with rare genital contact.  Patients were able to talk about their sexual relationships, and sexual interests did not cease.  Most could describe safe sex practices, but few followed it.  Lack of availability of condoms were identified. |
| 4 | Perlin, 2008 | Forensic inpatients, Asia | Not applicable | Discussion paper | ** | Poor | Described fear of adverse publicity, fear of litigation, sexual coercion, social attitude of sanism, an irrational prejudice against people with mental illness.  Civil rights while institutionalized was impacted. |

**Supplementary Table 1**

| Paper  Number | Author | Setting | Participants | Methods | Quality | Rating | Findings |
| --- | --- | --- | --- | --- | --- | --- | --- |
| 5 | Bartlett, 2010 | Forensic inpatients, UK | Forensic services  N = 39 | Qualitative | ** | Fair | Policies extremely controlled institutional life, by prohibiting or actively discouraging sexual relationships. Legal authority governed sexual and emotional expression. |
| 6 | Mercer, 2013 | Forensic inpatients and staff, UK | Mental health staff  N = 18  Forensic patients  N = 9 men | Qualitative | ** | Fair | The findings focus on performative language use; men's language contributed to a dominant masculine culture that reproduced gendered power relations and inequalities in a parallel way to evidence, identifying pornography as a public health concern in contemporary culture  Pornography could be a mechanism for achieving sexual satisfaction. Nurses were sympathetic to patient needs |
| 7 | Dolan, 2013 | Forensic inpatients, UK | Forensic patients  N = 225 women | Quantitative | * | Poor | High prevalence of childhood physical and sexual abuse and inter partner physical abuse |
| 8 | Brown, 2014 | Forensic inpatients, UK | Forensic patients  N = 20  n= 15 men  n= 5 women | Qualitative | ***** | Good | 30% of inpatients engage in sexual activity. Finding identified relationship difficulties, psychotropic medication side effects and lack of privacy, with excessive monitoring as factors for limited capacity for sexual expression during their stay in hospital  Unwillingness of staff to engage with sexual issues due to risk aversive practice. Other factors included Vulnerability and predation, ability of the patient to consent. |
| Paper Number | Author | Setting | Participants | Methods | Quality | Rating | Findings |
| 9 | Quinn, 2015 | Forensic inpatients and staff, Australia | Mental health staff  N = 12  Forensic patients  N = 10  n = 6 men  n = 4 women | Qualitative | **** | Good | Study described difficulties establishing sexual relationships, with lack of privacy, risks of  predatory sexual behaviours, consequences with relationships break downs, possibility of violence and sexually transmitted diseases.  Findings Identified lack of support, lack of sexual health discussion. |
| 10 | Quinn, 2015 | Forensic inpatients and staff, Australia | Mental health staff  N =12  Forensic patients  N = 10  n = 6 men  n = 4 women | Qualitative | **** | Good | Patients were sexually active often in secrecy. Masturbation was an accepted practice.  Study identified the need for a private and dignified place for patient intimacy  Lack of sexual discussions, over-emphasis on risk reduction were evident.  Institutional rules and rule adherence create barriers for patients. Sexual relationships are considered part of being human, yet they are prohibited. |
| 11 | Quinn, 2015 | Forensic inpatients and staff, Australia | Mental health staff  N = 12  Forensic patients  N = 10  n = 6 men  n = 4 women | Qualitative | *** | Good | Sexual identity issues, gender identity issues were prevalent.  Male on male sex without identifying as homosexual was noted.  Lack of sex education and training, no access to condoms, contributed to risk of abuse and pregnancy. |

| Paper Number | Author | Setting | Participants | Methods | Quality | Rating | Findings |
| --- | --- | --- | --- | --- | --- | --- | --- |
| 12 | Henrichs, 2015 | Forensic inpatients and community patients Nederland | Forensic patients  N =119  n = 101 men  n = 18 women | Quantitative | * | Poor | 32% forensic patient were married.  Perpetrators of interpersonal violence had higher rates of previous physical victimization. |
| 13 | Dein, 2016 | Forensic inpatients and staff, UK | Mental health staff  N = 24 | Qualitative | **** | Good | Discrimination against transgender and same sex relationships were reported.  Exploitation of vulnerable patients and fear of unwanted pregnancy were highlighted.  Clinical care in the inpatient setting had an absence of discourse on patient sexuality with sexuality rarely discussed.  Staff accept sexual desire as normal in asymptomatic and rehabilitation patients. Professionals used personal judgment to reach decisions on patient sexual activity.  No clear policy on the matter of patient sexuality |
| 14 | Quinn, 2016 | Forensic inpatients and staff, Australia | Mental health staff  N = 12  Forensic patients  N = 10  n = 6 men  n = 4 women | Qualitative | **** | Good | Unmet needs identified as support for sexual intimacy needs, privacy and opportunity.  Negative attitudes and responses by nurses were noted.  Nurses accept sexual relationships for patients in long-term units and saw an important role for nurses in providing support and an opportunity to work more closely with patients regarding sexual intimacy needs.. |
| Paper Number | Author | Setting | Participants | Methods | Quality | Rating | Findings |
| 15 | Tiwana, 2016 | Forensic inpatients, staff, Europe | Not applicable | Qualitative | ** | Fair | No country had a national policy, many had local policies or shared practices.  The UK appeared the most prohibiting and excluding, its protocols were apparently based on risk aversion and lack of emphasis or consideration of patients’ sexual needs.  Progressive approaches to patient sexuality were evident in nine of the countries. |
| 16 | Dein, 2018 | Forensic inpatients, UK | Not applicable | Discussion paper | **** | Good | Identified barriers for expressing health sexuality as lack of private spaces, lack of interpersonal skills, social withdrawal, medication side-effects and mental health symptoms, inadequate staffing and lack of training in staff about patient sexuality.  Risks outlined were the patient’s capacity to consent; management of allegations of rape; sexual exploitation of vulnerable patients; unsafe sexual practices; the trading of sex for money and drugs; the spread of sexually transmitted diseases; and unexpected pregnancies; public disapproval and negative media responses. |

| Paper Number | Author | Setting | Participants | Methods | Quality | Rating | Findings |
| --- | --- | --- | --- | --- | --- | --- | --- |
| 17 | Searle, 2018 | Forensic inpatients, UK | Forensic patients  N = 10 men | Qualitative | * | Poor | Study posited men to have pro-social explanatory frameworks for their representation of masculinity.  This constructed the ward as an alienated masculine territory characterized by a sexual division of labour that marginalized female nurses. |
| 18 | Huband, 2018 | Forensic inpatients, Europe | Not applicable | Qualitative | *** | Fair | The study Acknowledged impact on quality of life on forensic patient.  Recommended improving social skills and understanding of sexual experiences.  Study concluded at the time, there were consensus on what might constitute “best practice”. |
| 19 | Ravenhill, 2020 | Forensic inpatients and staff, UK | Mental health staff  N = 10  Forensic patients  N = 16  n = 10men  n = 6 women. | Qualitative | **** | Good | Sexual expression was conceptualised as organisational misbehaviour.  The UK have no formal policies to inform the management of inpatient sexuality, prohibitive approaches are favoured owing to risks of vulnerability and predation in this cohort of patients. |

| Paper Number | Author | Setting | Participants | Methods | Quality | Rating | Findings |
| --- | --- | --- | --- | --- | --- | --- | --- |
| 20 | Brand, 2021 | Forensic inpatients and community patients, Australia | Forensic patients  N = 4 men | Case reports | **** | Good | Bi-directional impact of mental health on sexual health was highlighted.  Patients reported sexual dysfunction, decreased libido, anorgasmia etc.  Gaps in the identification and assessment of the sexuality and sexual health needs  Lack of identification and management of appropriate and safe sexual experiences was pointed out.  Clinical setting with prolonged periods in a confined and strictly regulated hospital environment, does not support or promote sexual experiences |
| 21 | Brand, 2022 | Forensic community patients, Australia | Forensic patients  N = 14  n = 11 men  n = 3 women | Qualitative | **** | Good | Few patients were partnered and had a stable relationship, with less sexually active.  Challenges in socialising and communication were reported. mental health symptoms and medication side effects were reported as significant barriers. Patients can communicate around sexuality. Patients identify the areas, such as knowledge about what “rights” they would have in a relationship, working on their communication skills, and regular medication reviews to maximize the treatment effect while minimizing side effects. Participants indicated Forensic mental Health Teams could support patient sexual health and wellbeing. |

*Study quality utilised the Agency for Healthcare Research and Quality (AHRQ) criteria for observational studies with a consensus-based weighting score (West et al., 2002). Quality indicators were defined according to integrative review methods and criteria (Brown et al., 2011, Christmals and Gross, 2017, Souza et al., 2010, Whittemore and Knafl, 2005)and included (a) sample size, (b) study design, (c) attempts to control for the risk of bias, (d) use of appropriate and standardised measures, (e) use of appropriate statistics, (f) quality of the presentation of the results, and (g) generalisability. The studies were rated on a scale of 1 to 5 (*) based on the report’s quality assessment. An overall rating of good, fair, or poor was allocated to each study based on the relevance to our research question.*

Brown, A., Lubman, D. I. & Paxton, S. J. 2011. Reducing sexually-transmitted infection risk in young people with first-episode psychosis.(Report). *International Journal of mental Health Nursing,* 20**,** 12.

Christmals, D. & Gross, J. 2017. An integrative literature review framework for postgraduate nursing research reviews. *J Res Med Sci,* 5.

Souza, M. T. D., Silva, M. D. D. & Carvalho, R. D. 2010. Integrative review: what is it? How to do it? *Einstein (São Paulo),* 8**,** 102-106.

West, S., King, V., Carey, T. S., Lohr, K. N., Mckoy, N., Sutton, S. F. & Lux, L. 2002. Systems to rate the strength of scientific evidence. *Evid Rep Technol Assess (Summ)***,** 1-11.

Whittemore, R. & Knafl, K. 2005. The integrative review: updated methodology. *J Adv Nurs,* 52**,** 546-53.
